# Design a software real-time operation platform for wave piercing catamarans motion control using linear quadratic regulator based genetic algorithm

**DOI:** 10.1371/journal.pone.0196107

**Published:** 2018-04-30

**Authors:** Lihua Liang, Jia Yuan, Songtao Zhang, Peng Zhao

**Affiliations:** College of Automation, Harbin Engineering University, Harbin, Nangang District 145, Harbin city, Heilongjiang Province, China; Southwest University, CHINA

## Abstract

This work presents optimal linear quadratic regulator (LQR) based on genetic algorithm (GA) to solve the two degrees of freedom (2 DoF) motion control problem in head seas for wave piercing catamarans (WPC). The proposed LQR based GA control strategy is to select optimal weighting matrices (Q and R). The seakeeping performance of WPC based on proposed algorithm is challenged because of multi-input multi-output (MIMO) system of uncertain coefficient problems. Besides the kinematical constraint problems of WPC, the external conditions must be considered, like the sea disturbance and the actuators (a T-foil and two flaps) control. Moreover, this paper describes the MATLAB and LabVIEW software plats to simulate the reduction effects of WPC. Finally, the real-time (RT) NI CompactRIO embedded controller is selected to test the effectiveness of the actuators based on proposed techniques. In conclusion, simulation and experimental results prove the correctness of the proposed algorithm. The percentage of heave and pitch reductions are more than 18% in different high speeds and bad sea conditions. And the results also verify the feasibility of NI CompactRIO embedded controller.

## 1 Introduction

After the middle of 20th century, with the development of various maritime transport lines, the exploitation of ocean resources and military utility, the requirements of ship performance are also changing. Military technology is in the stage of change, a new generation of ships will also use more high-speed ship technology. High-speed vessels, especially wave piercing catamarans (WPC) have drawn widely attention for their performances. Moreover, maneuverability and strike capability in high sea levels are required in modern sea warfare that means excellent seakeeping performance is required. WPC is characterized in that (i) The thin demi-hulls help to reduce the wave resistance, (ii) The width of demi-hulls accounts for 15%∼20% of the ship beam, which is more conducive to the high-speed navigation of WPC. Due to above advantages, WPC is very suitable for the use of offshore combat ships because of its voyageable character [1, 2].

It is believed that ship motion could be reduced by 70%∼80% with a well designed ride control system (RCS). However, the bad RCS brings some bad effects, such as undesired sounds and lift losses etc. In order to solve the WPC motion problems, many strategies have been studied by researchers from different countries. The ride control of high speed mono hull ship and WPC had been studied by Australian scholars since 1995 to 2013 [3–5]. The simulation test bed of 40m and 79m catamarans were built by Haywood et al. in 1995. The heave and pitch simulation curves could be seen in their paper. They demonstrated the importance of simulation. Davis et al. compared with motions computed by a high speed, fixed frame, time domain strip theory taking account of the effect of the T-foil and stern tab ride control system in 2003. The computational method was found to predict correctly the increase of response with vessel speed and its decrease as the sea direction moves towards the beam. Lavroff et al. provided experimental benchmark information relating to the wave slam loads on WPC ferries in 2013. A 2.5m hydro-elastic segmented catamaran model have been developed based on the 112m INCAT Tasmania WPC to establish the peak wave slamming loads acting on the full-scale vessel. The seakeeping performance of the 112m WPC in different wave directions and strong wind were investigated by model experiments in tanks and monitoring of the ship at seas by Ikeda et al. from Japan [6, 7]. A 90m WPC with a T-foil and two flaps was studied by me and my research team over the past few years. A linear quadratic regulator controller with a genetic algorithm, a linear quadratic Gaussian optimum control theory, a state feedback control method and a model predictive algorithm controller were used to reduce heave and pitch motion of the WPC [8–11]. Moreover, the imitation humpback whale T-foil [12] and a new measuring lift device for fin stabilizers [13] were also studied by us to build a ship ride control system.

The mathematical model of WPC and the control strategy have been determined in previous researches. Nevertheless, comprehensive software operation demonstration platform has not been built. It has many important advantages (i.e. the operation time can be reduced to a few seconds) for testing purposes, when a real experiment is expensive and potentially dangerous. Santos et al. [14], argued the field-programmable gate arrays (FPGAs)-based a multi-state fuzzy logic controller to achieve better position tracking performance than software-based soft real-time platforms. In [15], they described the usage of FPGAs for the design, simulation and implementation of parameters optimization of interval tye-2 fuzzy controllers. According to above mentioned, the software instruments are efficient and flexible to realize the purpose and a well human-computer interaction interface is built.

In this paper, the optimal linear quadratic regulator (LQR) based on genetic algorithm (GA) is designed to solve the two degrees of freedom (2 DoF) motion control problem in head seas for WPC. The choices of weighting matrices (Q and R) are the main point of the LQR problem [16]. Conventionally, the weighting matrices optimization method, such as the trail and error method [17] not only increases the difficulty but also consumes long time to find the global optimum solution. Hence, the GA [18], due to it provides powerful search mechanisms, is considered an rapid and convenient method to solve the optimization problem of this paper. The main contribution of this paper are building the MATLAB and LabVIEW software plats to simulate the reduction effects of WPC and selecting the real-time (RT) NI CompactRIO embedded controller to test the effectiveness of the actuators based on proposed techniques. Finally, some interesting results are obtained.

The structure is composed of 5 sections in the paper. Section 2 presents the mathematical model of 2 DoF motion of WPC using actuators-a T-foil and two flaps in head seas. Section 3 details the GA method for parameters optimization based LQR controller. Section 4 presents the experimental results and discussions throughout software implementations. Finally, the conclusion is given.

## 2 Mathematical model of WPC

**Fig 1:**
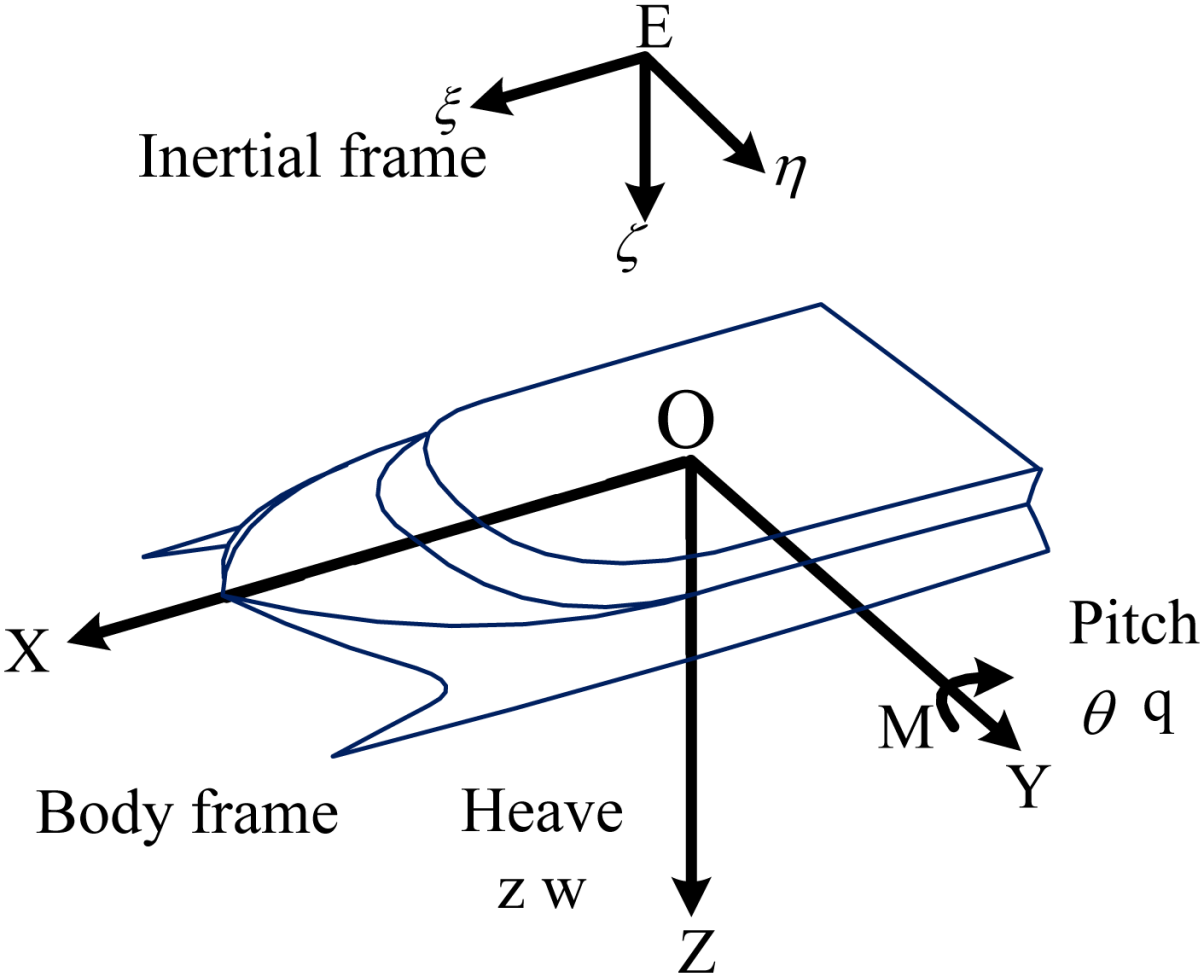
A 2 DoF WPC rigid body moving in a three-dimensional space.

There are 2 DoF motion for WPC when the ship is sailing in head seas, caused by two demi-hulls and high speeds: a movement around the *Z* axis namely heave, represented by the displacement *z*, and the rotation around the *Y* axis namely pitch, represented by the angle *θ*. The inertial frame is *E* − *ξηζ* and the corresponding linear and angular velocities in the body frame are denoted by *w* and $\dot{\theta }$. The system is a 2 DoF WPC rigid body moving in a three-dimensional space as shown in Fig 1. The kinematic equations are written in Eq (1), which describing the nonlinear problems with coupling terms [19–21].

$$\begin{array}{ccc} m_{z}\dot{w} & = & Z_{\dot{w}}\dot{w}+Z_{w}w+Z_{z}z+Z_{\dot{q}}\dot{q}+Z_{\dot{\theta }}\dot{\theta }+Z_{\theta }\theta +Z_{d}d+Z_{u}u\\ I_{yy}\dot{q} & = & M_{\dot{q}}\dot{q}+M_{\dot{\theta }}\dot{\theta }+M_{\theta }\theta +M_{\dot{w}}\dot{w}+M_{w}w+M_{z}z+M_{d}d+M_{u}u \end{array}$$ (1)

Where *Z* and *M* are forces in the *Z* direction and moments with respect to the *Y* axis, respectively, *m*_*z*_ is the effective masses of WPC in the body frame, *I*_*yy*_ is the moments of inertia with respect to the *Y* axis, the hydrodynamic coefficients are defined in this way: $Z_{z}=\frac{dZ}{dz}$, $Z_{w}=\frac{dZ}{dw}$, ⋯, $M_{u}=\frac{dM}{du}$, $Z_{\dot{w}}$, $Z_{\dot{q}}$, $M_{\dot{w}}$ and $M_{\dot{q}}$ denote the fluid inertia force coefficients, and $Z_{\dot{w}}$ denotes the added masses term, $Z_{\dot{q}}$ and $M_{\dot{w}}$ denote the added static moments term, and $M_{\dot{q}}$ denotes the added moments of inertia term, *Z*_*w*_, $Z_{\dot{\theta }}$, *M*_*w*_ and $M_{\dot{\theta }}$ denote the hydrodynamic damping term, *Z*_*z*_, *Z*_*θ*_, *M*_*z*_ and *M*_*θ*_ denote the coefficients of restoring force. The disturbing forces and torques *Z*_*d*_ and *M*_*d*_ in the *XYZ*-directions are caused by wave disturbances, *d* is the disturbance signal, and the control forces and moments *Z*_*u*_ and *M*_*u*_ are provided by a T-foil and two trim flaps, *u* is the control signal [22].

The calculation of hydrodynamic parameters in formula (1) is difficult and complex in different sea state numbers (SSN) and sailing speeds. Hence, we have to apply the strip theory [23] to solve the coefficients (see Table 1).

**Table 1:** The partial hydrodynamic parameters.

**No.**	**Encounter freq.rad/s**	$m_{z}-Z_{\dot{w}}$	*Z*_*w*_	*Z*_*z*_	$Z_{\dot{q}}$	$Z_{\dot{\theta }}$	*Z*_*θ*_
1	0.322	465.552	128.989	1723.105	-23739.068	10173.813	1395.221
2	0.379	418.639	143.126	1723.105	-18838.258	9202.067	1395.221
3	0.437	385.297	154.272	1723.105	-15213.711	8533.925	1395.221
4	0.495	359.214	165.644	1723.105	-12641.655	8041.491	1395.221
5	0.553	336.564	176.991	1723.105	-10742.748	7628.297	1395.221
⋯	⋯	⋯	⋯	⋯	⋯	⋯	⋯
**No.**	**Encounter freq.rad/s**	$I_{yy}-M_{\dot{q}}$	$M_{\dot{\theta }}$	*M*_*θ*_	$M_{\dot{w}}$	*M*_*w*_	*M*_*z*_
1	0.322	2077879.637	572926.02	733240.216	27601.008	-8986.222	1395.221
2	0.379	1386491.524	471845.038	733240.216	22077.353	-8027.241	1395.221
3	0.437	994971.11	397166.194	733240.216	17988.579	-7323.195	1395.221
4	0.495	752433.897	345998.778	733240.216	15162.4	-6742.15	1395.221
5	0.553	589995.096	309162.799	733240.216	13073.863	-6223.175	1395.221
⋯	⋯	⋯	⋯	⋯	⋯	⋯	⋯

Ocean waves in reality are extremely complex and they are usually irregular random waves. The irregular waves are given by the sum of a large number of essentially independent regular contributions with random phases, under the assumption of the linear theory [24, 25]. In this representation, the model of the seas elevation is given by:

$$\begin{array}{c} \zeta \left(t\right)=\sum_{i=1}^{n}\zeta_{i}\cos\left(\omega_{i}t+\varphi_{i}\right) \end{array}$$ (2)

Where the subscript *i* represents each component, *ω* is the wave frequency, (*rad*/*s*), *φ* is the random phase, (*rad*), the phase is chosen to be a stochastic variable with uniform distribution on the interval [0, 2*π*], *ζ* is the constant wave amplitude, the expression of wave amplitude is calculated by:

$$\begin{array}{c} \zeta =\sqrt{2S(\omega )\Delta \omega } \end{array}$$ (3)

Where Δ*ω* is the wave frequency spacing, (*rad*/*s*), *S*(*ω*) is the power spectral density (PSD), commonly referred to as the wave spectrum, (*m*^2^*s*). In this paper, International Towing Tank Conference (ITTC) one-parameter ocean wave spectrum (P-M spectrum) has been adopted and is given by the following expression:

$$\begin{array}{c} S\left(\omega \right)=\frac{8.1\times 10^{-3}g^{2}}{\omega^{5}}\exp\left(\frac{-3.11}{\omega^{4}h_{1/3}^{2}}\right) \end{array}$$ (4)

Where *g* = 9.81, (*m*/*s*^2^), *h*_1/3_ is the significant wave height and it is different in different sea conditions, (*m*).

In this paper, the disturbing forces *Z*_*d*_ and moments *M*_*d*_ are described by 91 regular wave superposition [26]. They can be calculated as following:

$$\begin{array}{ccc} Z_{d}\left(t\right) & = & \sum_{i=1}^{91}Z_{i}\cos\left(\omega_{i}t+\varphi_{i}\right)\\ M_{d}\left(t\right) & = & \sum_{i=1}^{91}M_{i}\cos\left(\omega_{i}t+\varphi_{i}\right) \end{array}$$ (5)

Where *Z*_*i*_ and *M*_*i*_ are the heave forces and pitch moments produced by the *i* th wavelet *ζ*_*i*_ disturbance to the hull, respectively.

**Fig 2:**
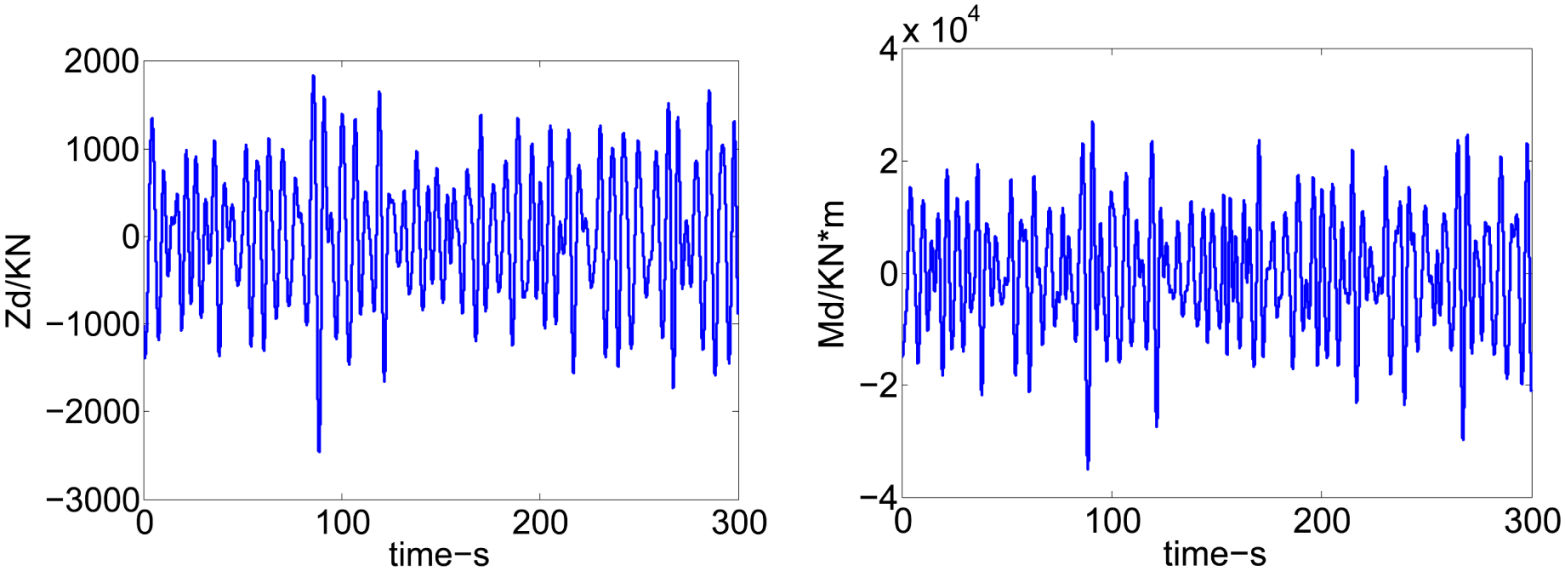
Disturbing forces and moments.

For practical reasons, the simulation resultants of heave disturbance forces and pitch disturbance moments are coded in MATLAB at SSN4, 40kn sailing speed and they are depicted in Fig 2.

**Fig 3:**
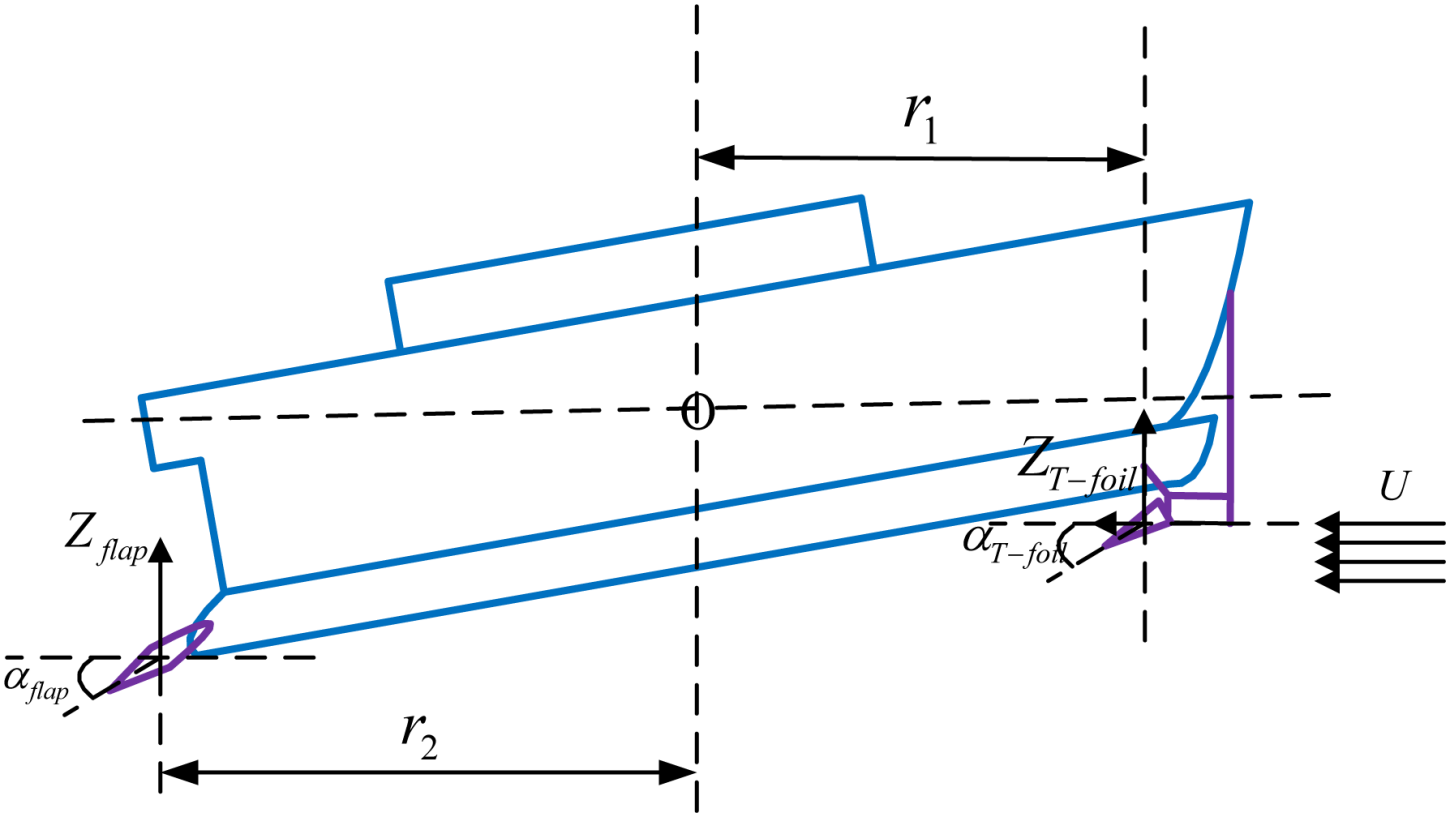
An anti-vertical motion system with actuators.

There is a T-foil located in the bow and two flaps located in the stern. Then, the displacements and angles of WPC in the heave and pitch axes directions are controlled by above actuators. The distance between the center of presser of the T-foil and the center of mass of the system is *r*_1_ and the distance between the center of presser of the flaps and the center of mass of the system is *r*_2_. Fig 3 is an anti-vertical motion system with actuators.

The stabilizers-induced forces and moments can be expressed as [27, 28]:

$$\begin{array}{ccc} Z_{u} & = & \frac{1}{2}\rho AU^{2}C_{L}\left(\alpha_{u}\right)\\ M_{u} & = & Z_{u}\cdot r \end{array}$$ (6)

Where *Z*_*u*_ = [*Z*_*T*−*foil*_, *Z*_*flap*_]^*T*^ and *M*_*u*_ = [*M*_*T*−*foil*_, *M*_*flap*_]^*T*^ are mentioned in above Eq (1), *ρ* is the density of water, (*kg*/*m*^3^), *A* is the area of T-foil or flaps, (*m*^2^), *U* is the speed of WPC, (*m*/*s*), *α*_*u*_ = [*α*_*T*−*foil*_, *α*_*flap*_]^*T*^ denotes the effective attack angle of T-foil and flaps, (*deg*), the actuators can rotate in −15° ∼ 15° and in −15° ∼ 0° respect to the horizontal, respectively, *C*_*L*_(*α*_*u*_) is the lift coefficient of the actuators obtained by computational fluid dynamics (CFD) software-FLUENT [29, 30], *r* = [*r*_1_, *r*_2_]^*T*^ are the distances between the center of pressure of above actuators and the center of mass of the system, (*m*).

## 3 Design of linear quadratic regulator based genetic algorithm

Linear quadratic regulator (LQR) is an optimal control strategy with the quadratic performance indexes and it is widely applied to the active control of deterministic vibratory systems. There are optimal multivariable feedback gains can be obtained by optimizing some predetermined performance criteria with LQR technique [31–33].

This is a controllable multi-input multi-output (MIMO) linear time-invariant (LTI) system so the kinematic Eq (1) can be rewritten in state-space expression [34, 35]. The formula is as follows:

$$\begin{array}{ccc} & & \left[\begin{array}{cc} m_{z}-Z_{\dot{w}} & Z_{\dot{q}}\\ M_{\dot{w}} & I_{yy}-M_{\dot{q}} \end{array}\right]\left[\begin{array}{c} \dot{w}\\ \dot{q} \end{array}\right]+\left[\begin{array}{cc} Z_{w} & Z_{\dot{\theta }}\\ M_{w} & M_{\dot{\theta }} \end{array}\right]\left[\begin{array}{c} w\\ \dot{\theta } \end{array}\right]+\left[\begin{array}{cc} Z_{z} & Z_{\theta }\\ M_{z} & M_{\theta } \end{array}\right]\left[\begin{array}{c} z\\ \theta \end{array}\right]\\ & & \;=\left[\begin{array}{cc} Z_{d} & Z_{u}\\ M_{d} & M_{u} \end{array}\right]\left[\begin{array}{c} d\\ u \end{array}\right] \end{array}$$ (7)

*S.t.* $\left[A_{a}\right]=\left[\begin{array}{cc} m_{z}-Z_{\dot{w}} & Z_{\dot{q}}\\ M_{\dot{w}} & I_{yy}-M_{\dot{q}} \end{array}\right]$, $\left[B_{b}\right]=\left[\begin{array}{cc} Z_{w} & Z_{\dot{\theta }}\\ M_{w} & M_{\dot{\theta }} \end{array}\right]$, $\left[C_{c}\right]=\left[\begin{array}{cc} Z_{z} & Z_{\theta }\\ M_{z} & M_{\theta } \end{array}\right]$.

Then,

$$\begin{array}{c} \left[\begin{array}{c} \dot{w}\\ \dot{q}\\ w\\ \dot{\theta } \end{array}\right]=\left[\begin{array}{cc} -A_{a}^{-1}B_{b} & -A_{a}^{-1}C_{c}\\ I_{2\times 2} & 0_{2\times 2} \end{array}\right]\left[\begin{array}{c} w\\ \dot{\theta }\\ z\\ \theta \end{array}\right]+\left[\begin{array}{c} A_{a}^{-1}\\ 0_{2\times 2} \end{array}\right]\left[\begin{array}{c} Z_{d}\\ M_{d} \end{array}\right]d+\left[\begin{array}{c} A_{a}^{-1}\\ 0_{2\times 2} \end{array}\right]\left[\begin{array}{c} Z_{u}\\ M_{u} \end{array}\right]u \end{array}$$ (8)

Where state variables $x={\left[w,\dot{\theta },z,\theta \right]}^{T}$, output variables *y* = *x*, *d* is the disturbance signal inputs and *u* is the control signal inputs. *S.t.* $A=[\begin{array}{cc} -A_{a}^{-1}B_{b} & -A_{a}^{-1}C_{c}\\ I_{2\times 2} & 0_{2\times 2} \end{array}]$, $B_{1}=\left[\begin{array}{c} A_{a}^{-1}\\ 0_{2\times 2} \end{array}\right]\left[\begin{array}{c} Z_{d}\\ M_{d} \end{array}\right]$, $B_{2}=\left[\begin{array}{c} A_{a}^{-1}\\ 0_{2\times 2} \end{array}\right]\left[\begin{array}{c} Z_{u}\\ M_{u} \end{array}\right]$.

Finally, the state-space equation of this system is expressed as:

$$\begin{array}{ccc} \dot{x} & = & Ax+B_{2}u+B_{1}d\\ y & = & Cx \end{array}$$ (9)

Where *A* ∈ ℜ^4×4^ is the system matrix, *B*_2_ ∈ ℜ^4×2^ is the control matrix, *B*_1_ ∈ ℜ^4×2^ is the wave disturbance matrix, *C* = *I*_4×4_ ∈ ℜ^4×4^ is the output matrix.

And then, we have to get the control inputs *u* to minimize following performance index [16, 32–34]:

$$\begin{array}{c} J=\frac{1}{2}\int_{0}^{\infty }\left(x^{T}Qx+u^{T}Ru\right)dt \end{array}$$ (10)

Where *Q* ∈ ℜ^4×4^ and *R* ∈ ℜ^2×2^ are weight matrices, *Q* is assumed to be a semi-positive definite matrix on the state penalty and *R* is assumed to be a positive definite matrix on the control penalty [36]. Both *Q* and *R* should be symmetric matrices and they are selected as the diagonal matrices usually.

The control inputs *u* can be written as:

$$\begin{array}{c} u=-Kx \end{array}$$ (11)

Where *K* is a desired gain and it is calculated as:

$$\begin{array}{c} K=R^{-1}B_{2}^{T}P \end{array}$$ (12)

Where *P* is the solution of Algebraic Riccati Equation (ARE) and the following equation must be satisfied:

$$\begin{array}{c} PA+A^{T}P-PB_{2}R^{-1}B_{2}^{T}P+Q=0 \end{array}$$ (13)

According to Eqs (11)–(13), the optimal control inputs are obtained by choosing proper weighting matrices *Q* and *R* to calculate the ARE. Traditionally, the weighting matrices can be decided with the error and trail approach [34]. However, there is always uncertainty due to human error. Intuitively, it is a time-consuming work until the final optimum control is reached.

Thus, genetic algorithm (GA) is found to obtain the desired gain *K* and it is the progress of manual mode. GA is one of the artificial intelligence (AI) techniques [18] in sets of evolutionary computations. Depending on robust search mechanisms and global optimum, GA is applied widely in plenty of optimum control system fields [37].

Main processes for applying GA to obtain a best heave and pitch effects of WPC are presented [16, 35, 37].

### Step 1 encoding

The real-coded genetic algorithm (RCGA) is applied in this study. Therefore, the *Q* and *R* are coded as:

$$\begin{array}{c} \Lambda =(x_{1},x_{2},x_{3},x_{4},x_{5},x_{6}) \end{array}$$ (14)

Where *x*_1_, *x*_2_, *x*_3_ and *x*_4_ are the weights of velocity of heave, velocity of pitch, heave and pitch, respectively. Similarly, *x*_5_ and *x*_6_ are the weights of two control inputs and they are two different attack angles of a T-foil and two flaps. As a result, the values of *Q* and *R* are related to their contribution to the performance index *J*.

Hence, Eq (10) can be rewritten as:

$$\begin{array}{c} J=\frac{1}{2}\int_{0}^{\infty }\left(x_{1}w^{2}+x_{2}\dot{\theta^{2}}+x_{3}z^{2}+x_{4}\theta^{2}+x_{5}\alpha_{T-foil}^{2}+x_{6}\alpha_{flap}^{2}\right)dt \end{array}$$ (15)

### Step 2 choosing fitness function

The final responses of WPC ride control system (RCS) are determined by following fitness function:

$$\begin{array}{c} J\left(\kappa \right)=\min_{\kappa \in S}\left[d_{1}\cdot \left(\frac{1}{N}\sum_{i=1}^{N}w^{2}\right)+d_{2}\cdot \left(\frac{1}{N}\sum_{i=1}^{N}{\dot{\theta }}^{2}\right)+d_{3}\cdot \left(\frac{1}{N}\sum_{i=1}^{N}z^{2}\right)+d_{4}\cdot \left(\frac{1}{N}\sum_{i=1}^{N}\theta^{2}\right)\right] \end{array}$$ (16)

Where *κ* is parameter vector, *S* is span of parameter vector, *N* is the maximum number of generations, *d*_*i*_, *i* = 1, 2, 3, 4 is used to balance the control effect of each output variable, through the comparison of different values of *d*_*i*_, the optimal results are obtained, *w*, $\dot{\theta }$, *z* and *θ* are described in Section 2.

### Step 3 generating initial population and the next generation

Firstly, the initial population starts from random population, and then the next generation is obtained by the random population through the selection of elite children, crossover (mating), and mutation process. In each generation, the fitness function of every individual is evaluated, as well as some individuals are stochastically selected and modified from the current population to generate a new population. Hence, the new population is used in the next iteration process of GA.

### Step 4 iterating termination condition

The stop algebra is applied to check the iteration termination when either the maximum number of generations *N* is produced, or a satisfactory fitness value is reached in Step 2. If true, exit the GA. Otherwise, go back to Step 3.

**Fig 4:**
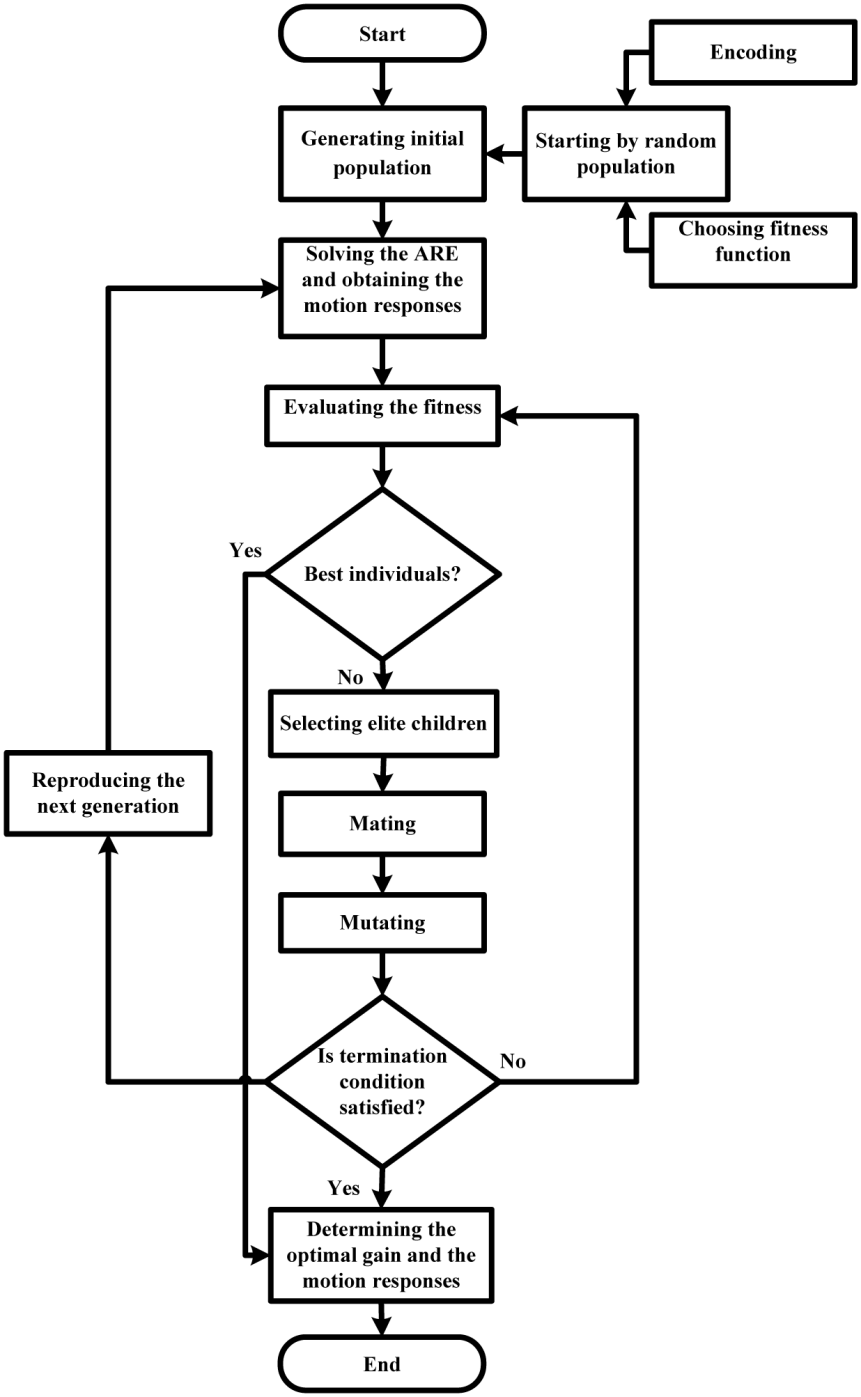
Steps of GA based LQR.

**Fig 5:**
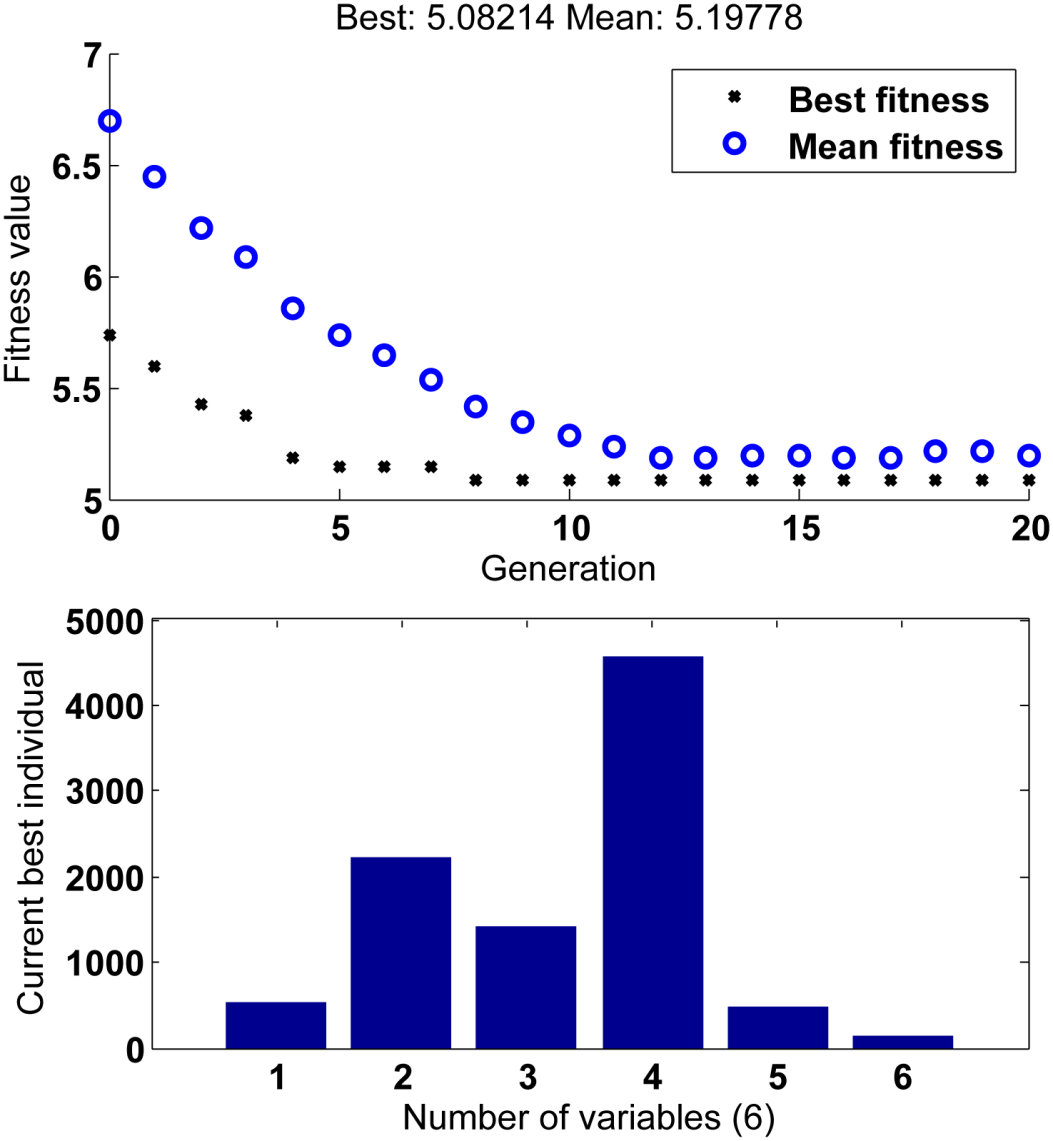
The optimum GA results.

GA is implemented using simulation software in man-machine interactive way. The settings of GA are outlined in Table 2. All of above steps are shown in Fig 4 [16, 32, 35]. Fig 5 shows the optimum GA results, where the value of fitness curve is the result of fitness function (16) and the current best individual is the values of the weighting matrices *Q* and *R*. Consequently, the results of weighting matrices *Q* and *R*, the solution of ARE *P* and the desired gain *K* are listed in Table 3.

**Table 2:** GA parameters settings.

GA Parameters	Value
Population size	100
Elitism	10
Crossover	0.4
Upper and lower bounds	*LB* = [1, 1, 1, 1, 1, 1] *UB* = [5000, 5000, 5000, 5000, 500, 500]
Mutation	20
Max iterations	20

**Table 3:** The results of LQR-GA.

Parameters of system	LQR-GA
Weighting matrix (*Q*)	$\left[\begin{array}{cccc} 535.5954 & 0 & 0 & 0\\ 0 & 2227.4 & 0 & 0\\ 0 & 0 & 1415.8 & 0\\ 0 & 0 & 0 & 4565.5 \end{array}\right]$
Weighting matrix (*R*)	$\left[\begin{array}{cc} 492.9223 & 0\\ 0 & 154.9472 \end{array}\right]$
Solution of ARE (*P*)	$\left[\begin{array}{cccc} 1.7501 & -5.2546 & 2.6010 & -5.0355\\ -5.2546 & 100.4556 & -5.1423 & 98.4886\\ 2.6010 & -5.1423 & 876.1099 & -20.6821\\ -5.0355 & 98.4886 & -20.6821 & 3514.6 \end{array}\right]$
Optimal gain (*K*)	$\left[\begin{array}{cccc} -0.9983 & -0.5883 & -1.5967 & -0.6578\\ 0.5428 & -3.9510 & 0.7336 & -3.8472 \end{array}\right]$

**Proof.** Proof of stability of the controller based on genetic algorithms

Select the following Lyapunov function:

$$\begin{array}{c} V\left(x\right)=x^{T}Px\left(V\left(x\right)>0,\forall x\neq 0;V\left(x\right)\to \infty ,\forall ∥x∥\to \infty \right) \end{array}$$ (17)

Where *P* is a positive definite symmetric solution of ARE (13). The first-order time derivative of *V*(*x*) is:

$$\begin{array}{c} \dot{V}\left(x\right)={\dot{x}}^{T}Px+x^{T}P\dot{x} \end{array}$$ (18)

Substituting Eqs (9), (11), (12) and (13) into Eq (18) yields:

$$\begin{array}{ccc} \dot{V}\left(x\right) & = & {\left[\left(A-B_{2}R^{-1}B_{2}^{T}P\right)x\right]}^{T}Px+x^{T}P\left(A-B_{2}R^{-1}B_{2}^{T}P\right)x\\ & = & x^{T}\left(A^{T}-P^{T}B_{2}{\left(R^{-1}\right)}^{T}B_{2}^{T}\right)Px+x^{T}PAx-x^{T}PB_{2}R^{-1}B_{2}^{T}Px\\ & = & x^{T}A^{T}Px-x^{T}P^{T}B_{2}{\left(R^{-1}\right)}^{T}B_{2}^{T}Px+x^{T}PAx-x^{T}PB_{2}R^{-1}B_{2}^{T}Px\\ & = & x^{T}\left(A^{T}P-P^{T}B_{2}{\left(R^{-1}\right)}^{T}B_{2}^{T}P+PA-PB_{2}R^{-1}B_{2}^{T}P\right)x\\ & = & x^{T}\left(-Q-P^{T}B_{2}{\left(R^{-1}\right)}^{T}B_{2}^{T}P\right)x\\ & = & -x^{T}\left(Q+PB_{2}R^{-1}B_{2}^{T}P\right)x<0 \end{array}$$ (19)

According to the Lyapunov stability theorem [38, 39], the proposed controller based on genetic algorithms stabilizes the system given by Eq (9).

**Fig 6:**
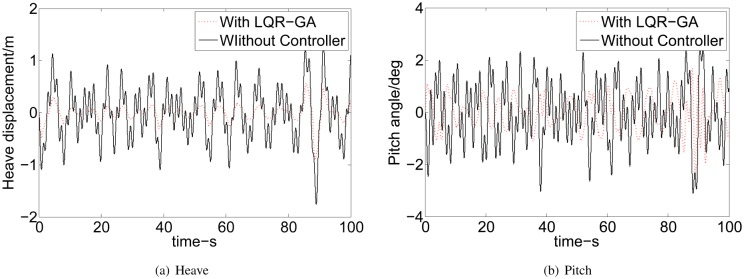
Effects of WPC ride control with LQR-GA approach on heave and pitch. (a) Heave. (b) Pitch.

Here our main aim is to reduce the heave displacement and pitch angle of WPC. Then, the effects of WPC ride control with LQR-GA approach are shown in Fig 6. It can be seen that the heave is reduced 65.33% on average and the pitch is reduced 44.81% on average when WPC is sailing at 40kn and SSN4. Obviously, the heave reduction is better than pitch. It is necessary to trade-off the reduction of heave and pitch. In this paper, the pitch reduction is good when we ensure that the heave reduction is also good. Meanwhile, the pitch reduction will be hysteretic.

## 4 Experimental results and discussions

### 4.1 System development and simulation using MATLAB environment

**Fig 7:**
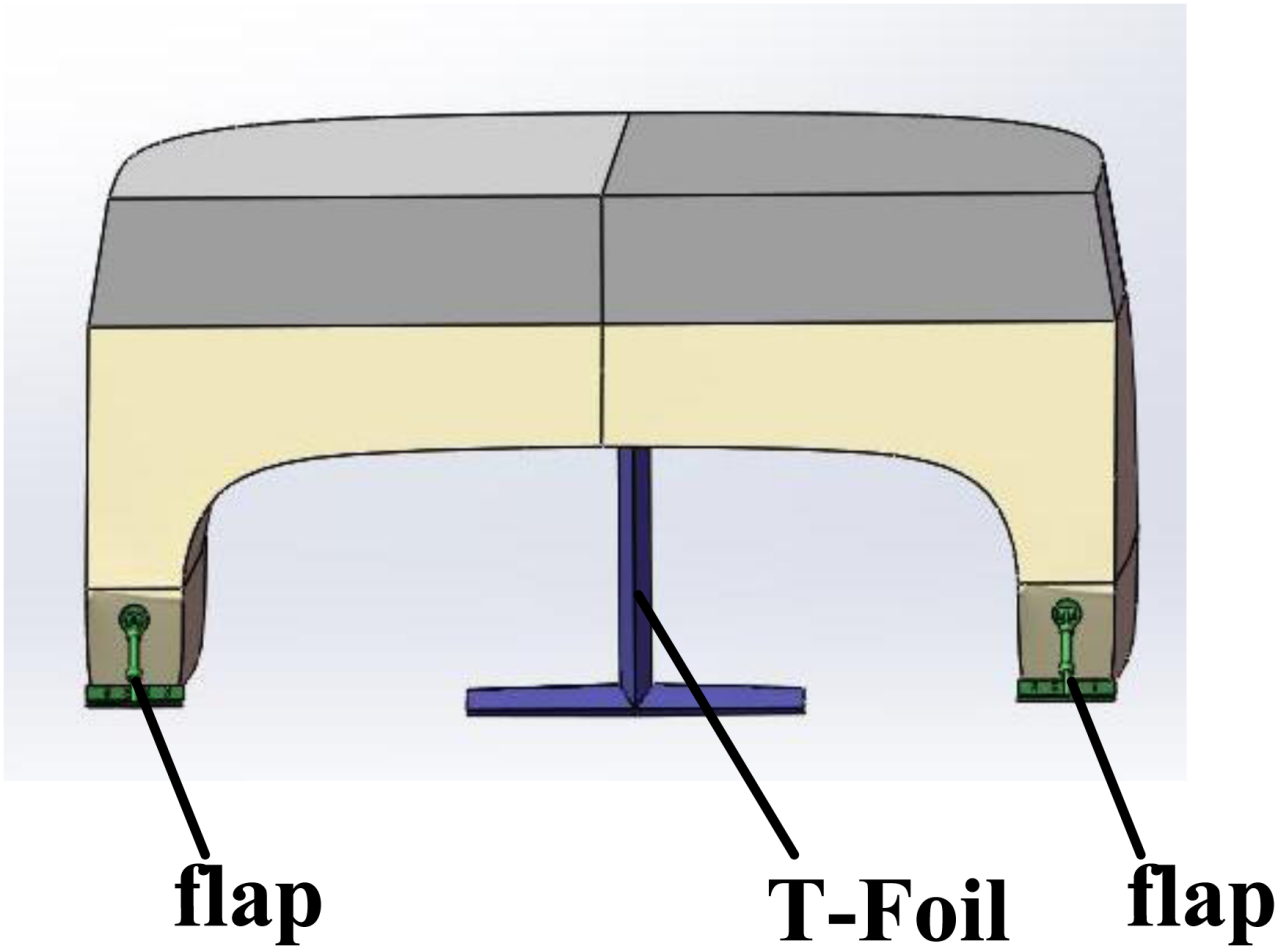
A WPC with a T-foil and two flaps.

The 2 DoF mathematical model is built and the LQR controller based GA is designed in the above stages. A 90 meters WPC equipped with a T-foil and two flaps is shown in Fig 7. Table 4 summarizes the main characteristics of WPC.

**Table 4:** Nominal physical parameters of WPC.

Parameters	Sign	Values	Unit
Length	*L*	90	m
Beam	*B*	25.96	m
Waterline Length	*LW*	85.655	m
Waterline Beam	*BW*	25.928	m
Draught	*D*	2.6	m
Displacement	Δ	734.541	ton
Transverse Metacenter Height	$\bar{GM}$	66.42	m

**Fig 8:**
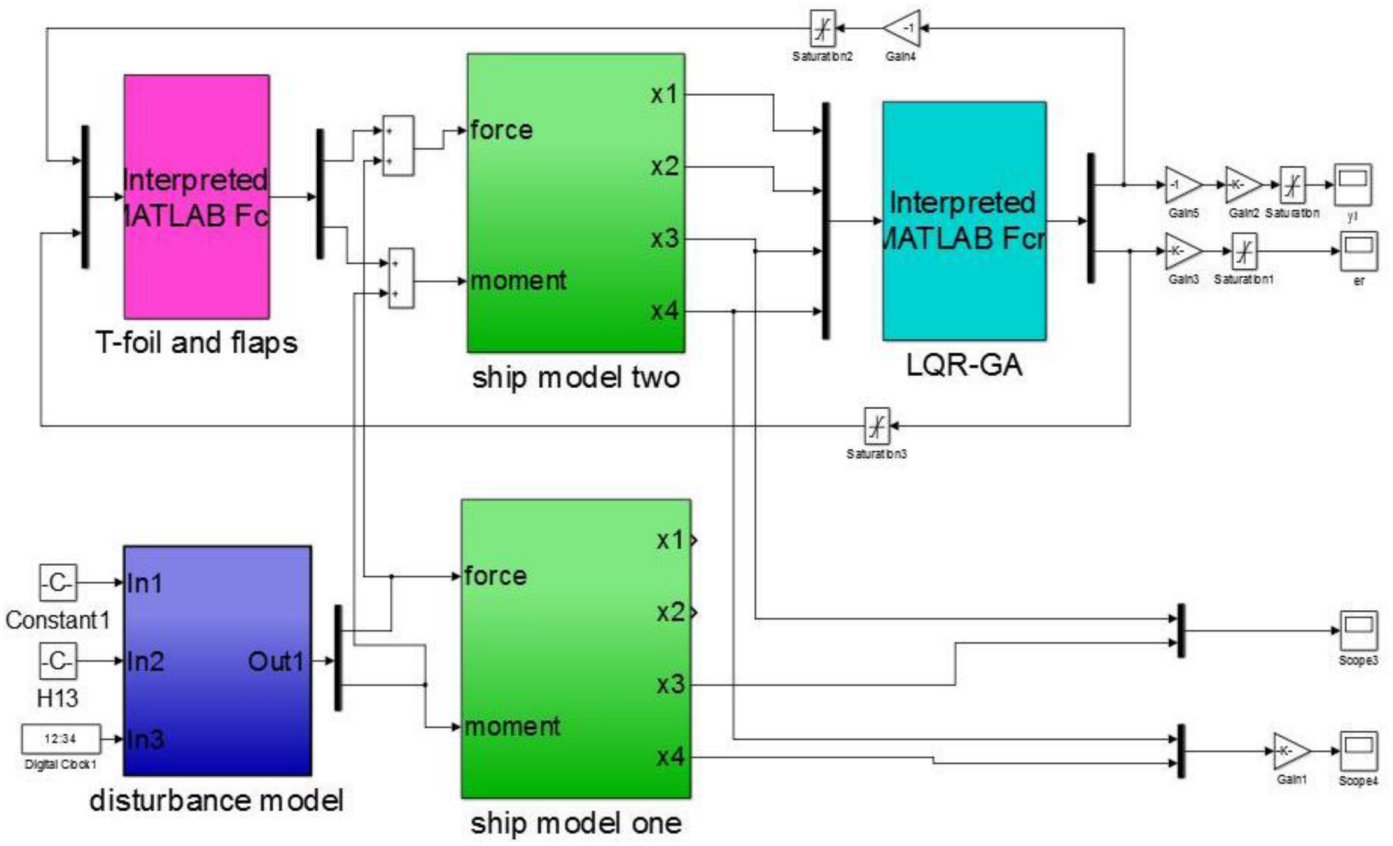
SIMULINK diagrams of the controlled WPC.

In general, a complete model in its operational environment includes a controlled plant, a appropriate controller, some related actuators and external perturbations. There is a expedient tool for control design by adding data input devices and display interfaces units. That is MATLAB provides a complete set of toolboxes for proposed control design. There are four major blocks as following SIMULINK diagram (see Fig 8). The block labelled “ship model” is an input/output model of the 2 DoF motion of WPC in response to waves (labelled as “ship model one”) and two actuators (labelled as “ship model two”). The block labelled “disturbance model” is a disturbance input can be coupled to the “ship model one” that is a model of WPC without control device. Opening the dialog window of “disturbance model”, the speed, the significant wave height and the wave encounter angle are selected as disturbed conditions to be simulated. The block labelled “T-foil and flaps” is a model of the actuators can be coupled to the “ship model two” that is a ship model with control devices. There are two limit control modules because of the effective flapping angles of two actuators mentioned in Section 2. The block labelled “LQR-GA” gets information from the behavior of WPC, and computes two control inputs, then the command signals of two actuators are obtained.

It can be seen that above model has two main components. One component obtains the heave forces and the pitch moments induced by the incident waves can be described as bare boat motion. While the other component obtains the forces and moments produced by two actuators can be coupled to the bare boat model with proposed control strategy.

**Fig 9:**
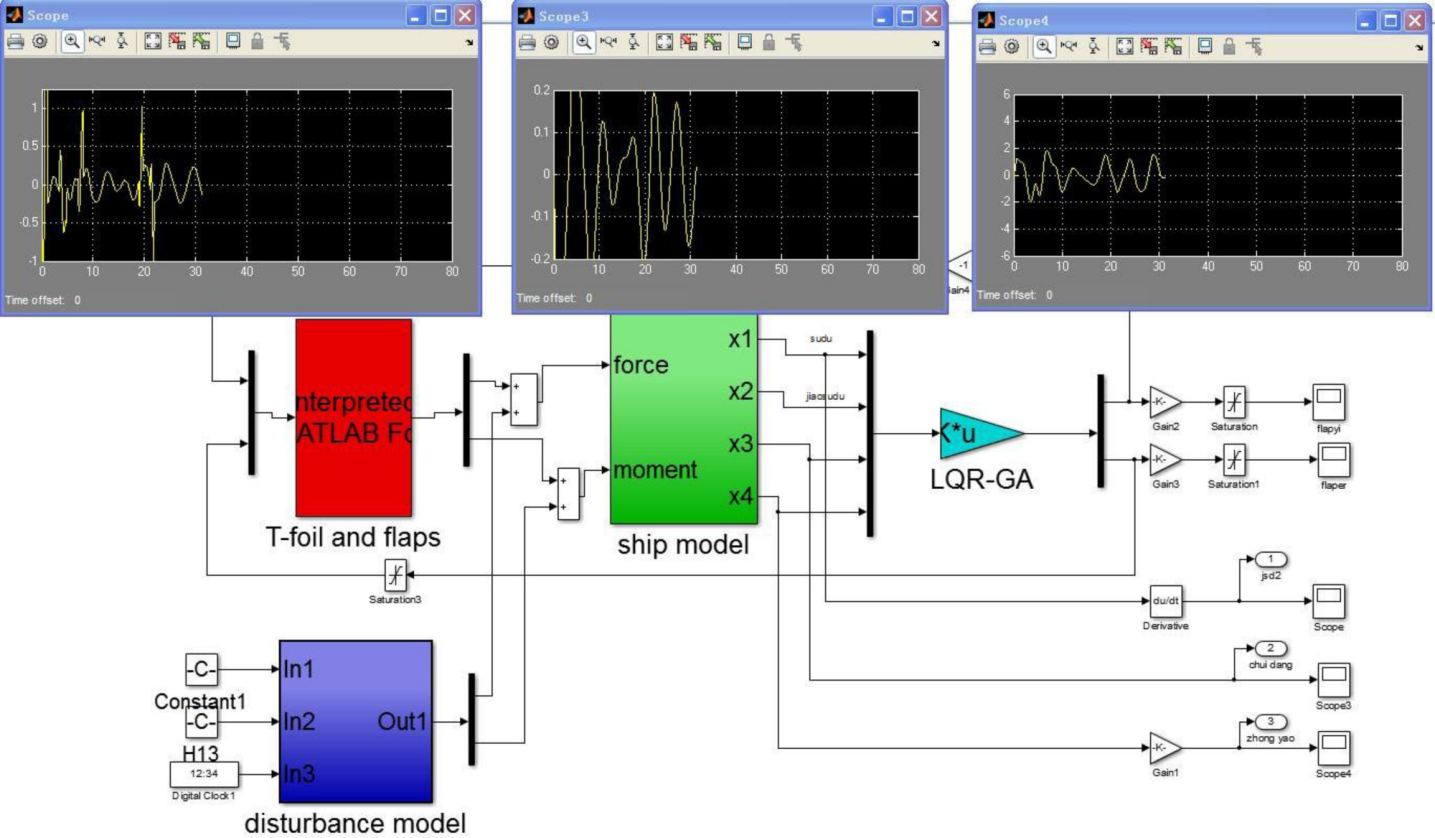
An interactive screen of GA SIMULINK diagram.

We have to tune the weighting matrices of the LQR using a computer by a systematic searching-GA. It is easy to implement a simulation environment by means of an interactive procedure (see Fig 9). Fig 9 shows a computer screen of the running simulation interface. The SIMULINK diagram modules are similar to Fig 8. There are also four major blocks refereed as “ship model”, “disturbance model”, “T-foil and flaps” and “LQR-GA”. The difference between them is “LQR-GA” module. The desired gain *K* is obtained by the background processing procedures using MATLAB script and the *Q* and *R* are obtained by optimizing the parameters of proposed algorithm. The optimal attack angles of the T-foil and flaps are obtained by iterative calculation to decrease the vertical motion. Above three windows in Fig 9 display three output variables can be observed a simulated journey. They are heave accelerations, heave displacements and pitch angles controlled by LQR-GA, respectively.

**Fig 10:**
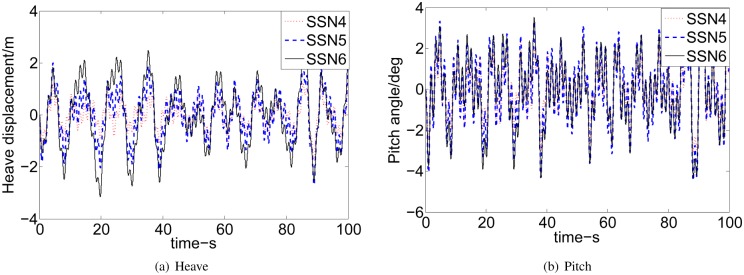
Comparison of effects at 40 knots. (a) Heave. (b) Pitch.

**Fig 11:**
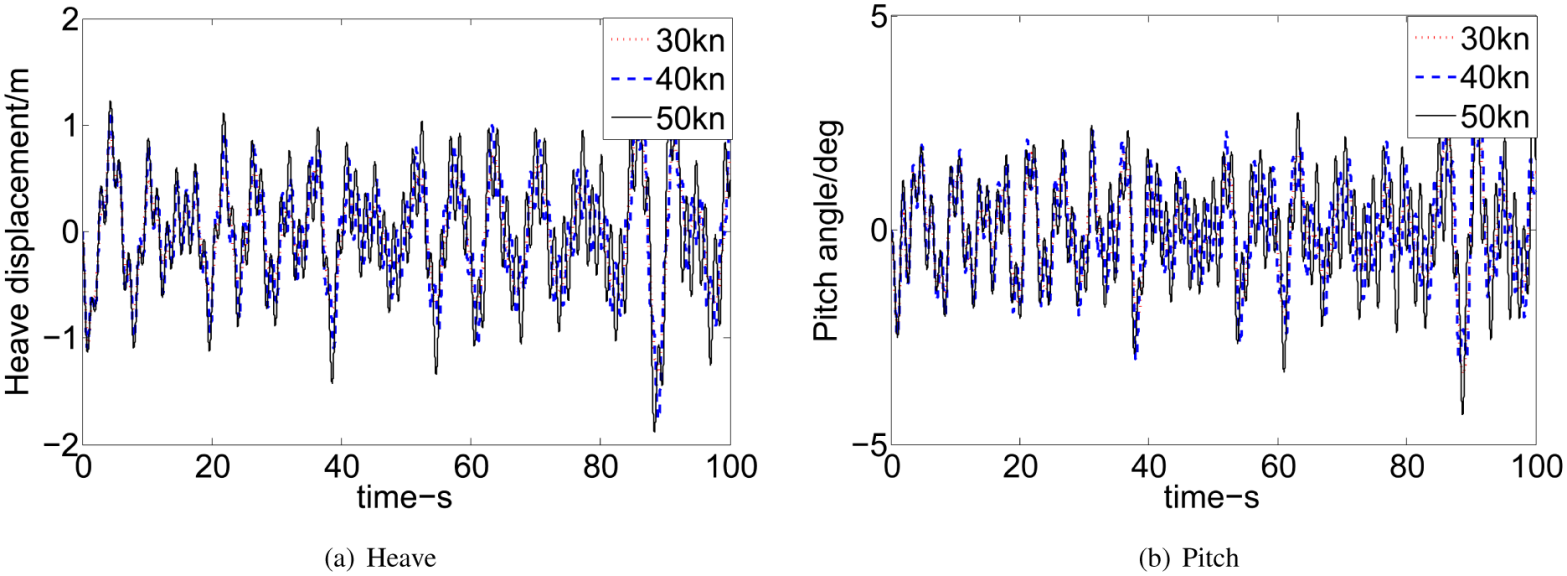
Comparison of effects at SSN4. (a) Heave. (b) Pitch.

**Fig 12:**
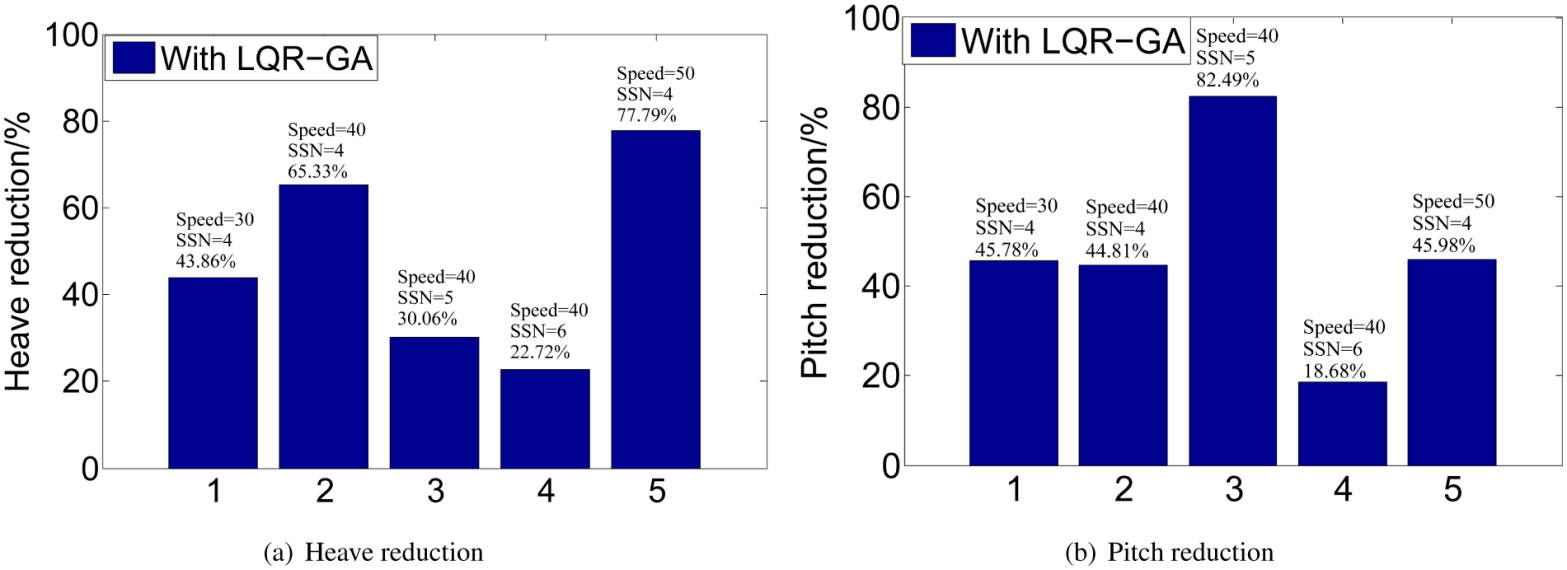
Comparison of vertical motion in five conditions. (a) Heave reduction. (b) Pitch reduction.

Figs 10 and 11 show that the heave and pitch are increased with the increase of SSN and speeds. It can be seen that our research interest focuses on SSN 4, 5, and 6 and ship’s speeds at 30, 40 and 50 knots. The MATLAB environment we have established can be easily tested for different control designs to get the different control solutions and can visualize the vertical motion of WPC. The proposed controller is tuned to achieve the best results of 2 DoF of WPC. Fig 12 shows the reduction percentage of vertical motion. The percentage of heave and pitch reductions are more than 18% in different high speeds and the bad sea conditions. While, the reduction of vertical motion are up to 70%∼80% under some specified conditions. And the weighting parameters and the desired gain in different SSN and speeds are listed in Table 5.

**Table 5:** The weighting parameters and the desired gain in different SSN and speeds.

SSN	Speeds	*Q*	*R*	*K*
4	40/kn	$\left[\begin{array}{cccc} 535.5954 & 0 & 0 & 0\\ 0 & 2227.4 & 0 & 0\\ 0 & 0 & 1415.8 & 0\\ 0 & 0 & 0 & 4565.5 \end{array}\right]$	$\left[\begin{array}{cc} 492.9223 & 0\\ 0 & 154.9472 \end{array}\right]$	$\left[\begin{array}{cccc} -0.9983 & -0.5883 & -1.5967 & -0.6578\\ 0.5428 & -3.9510 & 0.7336 & -3.8472 \end{array}\right]$
5	40/kn	$\left[\begin{array}{cccc} 2.25 & 0 & 0 & 0\\ 0 & 2455.3 & 0 & 0\\ 0 & 0 & 1 & 0\\ 0 & 0 & 0 & 2090.8 \end{array}\right]$	$\left[\begin{array}{cc} 7.8509 & 0\\ 0 & 120.6499 \end{array}\right]$	$\left[\begin{array}{cccc} -0.2201 & -16.8155 & -0.2253 & -14.2473\\ 0.1102 & -2.0003 & 0.0126 & -1.0633 \end{array}\right]$
6	40/kn	$\left[\begin{array}{cccc} 4995.6 & 0 & 0 & 0\\ 0 & 1485.1 & 0 & 0\\ 0 & 0 & 2184.7 & 0\\ 0 & 0 & 0 & 4924 \end{array}\right]$	$\left[\begin{array}{cc} 3.0372 & 0\\ 0 & 345.7177 \end{array}\right]$	$\left[\begin{array}{cccc} -40.4783 & -1.1615 & -26.7795 & -1.6024\\ 0.1835 & -2.4218 & 0.0007 & -2.5056 \end{array}\right]$
4	30/kn	$\left[\begin{array}{cccc} 597.1507 & 0 & 0 & 0\\ 0 & 911.0533 & 0 & 0\\ 0 & 0 & 1310.7 & 0\\ 0 & 0 & 0 & 1462.1 \end{array}\right]$	$\left[\begin{array}{cc} 460.1734 & 0\\ 0 & 97.1971 \end{array}\right]$	$\left[\begin{array}{cccc} -1.0759 & -0.4086 & -1.5441 & -0.2598\\ 0.8242 & -3.2286 & 0.9952 & -1.8217 \end{array}\right]$
4	50/kn	$\left[\begin{array}{cccc} 3397.6 & 0 & 0 & 0\\ 0 & 911.3033 & 0 & 0\\ 0 & 0 & 1311 & 0\\ 0 & 0 & 0 & 4924.5 \end{array}\right]$	$\left[\begin{array}{cc} 3.6009 & 0\\ 0 & 49.3162 \end{array}\right]$	$\left[\begin{array}{cccc} -2.5576 & -0.5369 & -1.5746 & -0.9398\\ 3.2111 & -4.2820 & 1.8962 & -6.8268 \end{array}\right]$

### 4.2 System development and simulation using LabVIEW environment

The LabVIEW (Laboratory Virtual Instrumentation Engineering Workbench) software is chosen as a human-computer interaction interface and it is a graphical user interfaces (GUI). The operator requests a command line interface and uses visual programming language to create programs in a “block diagram” form. It can be seen the detailed information of each part of the test plat when in the background program interface. The results can be observed in front display panel with a graphical form [40–42].

**Fig 13:**
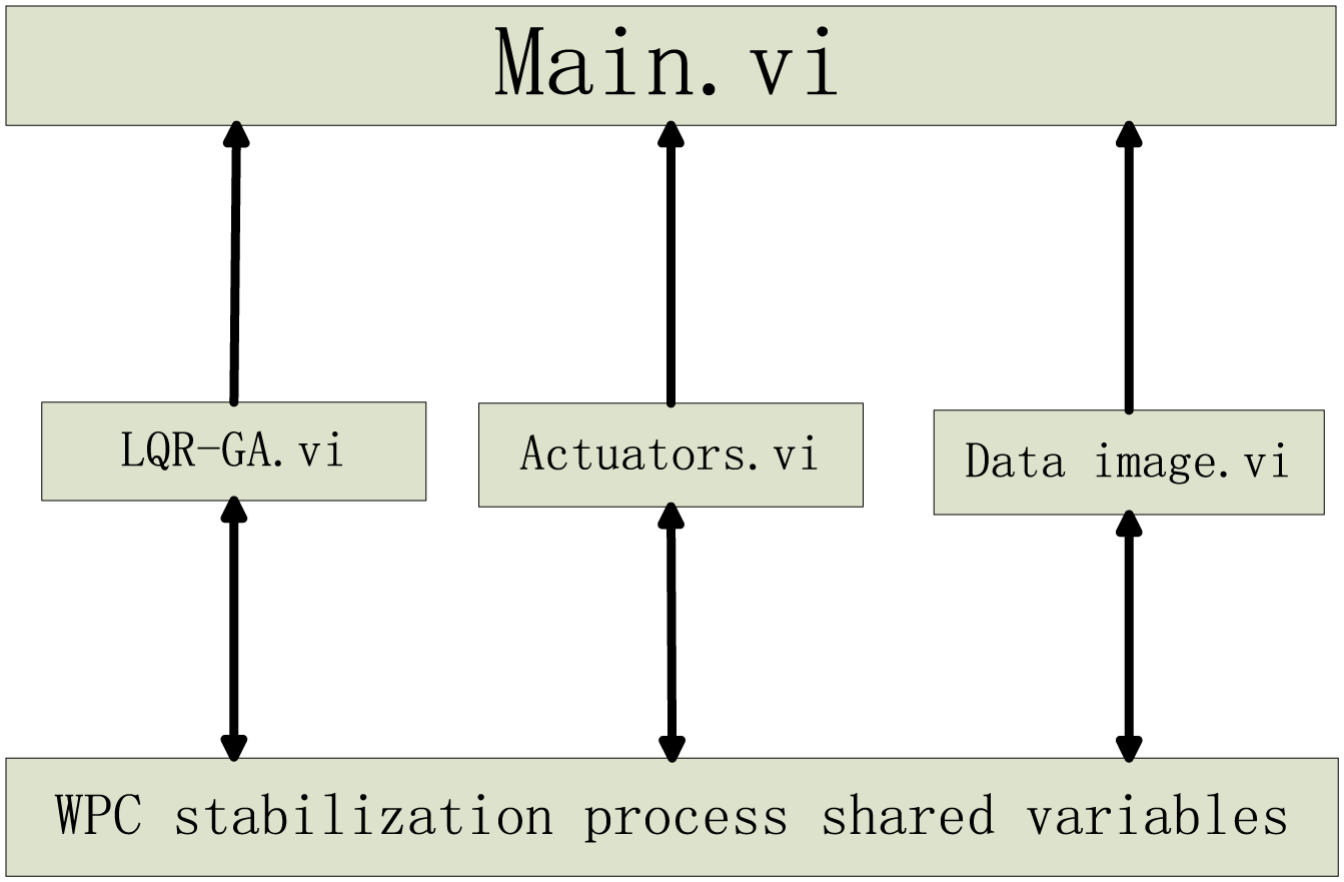
VI structure for WPC.

The functions of LabVIEW are expressed as virtual instruments (VIs). The VI structure of WPC stabilization process is shown in Fig 13. There are three components in each VI files: a program block diagram, a demo front panel, and a connector panel. LQR-GA.vi is the subroutine for proposed algorithm. The T-foil and flaps are handled in Actuators.vi. Data image.vi displays the WPC image and records ship motion data. Main.vi is put in charge of all subroutine VIs. Shared variables are included in the above files.

**Fig 14:**
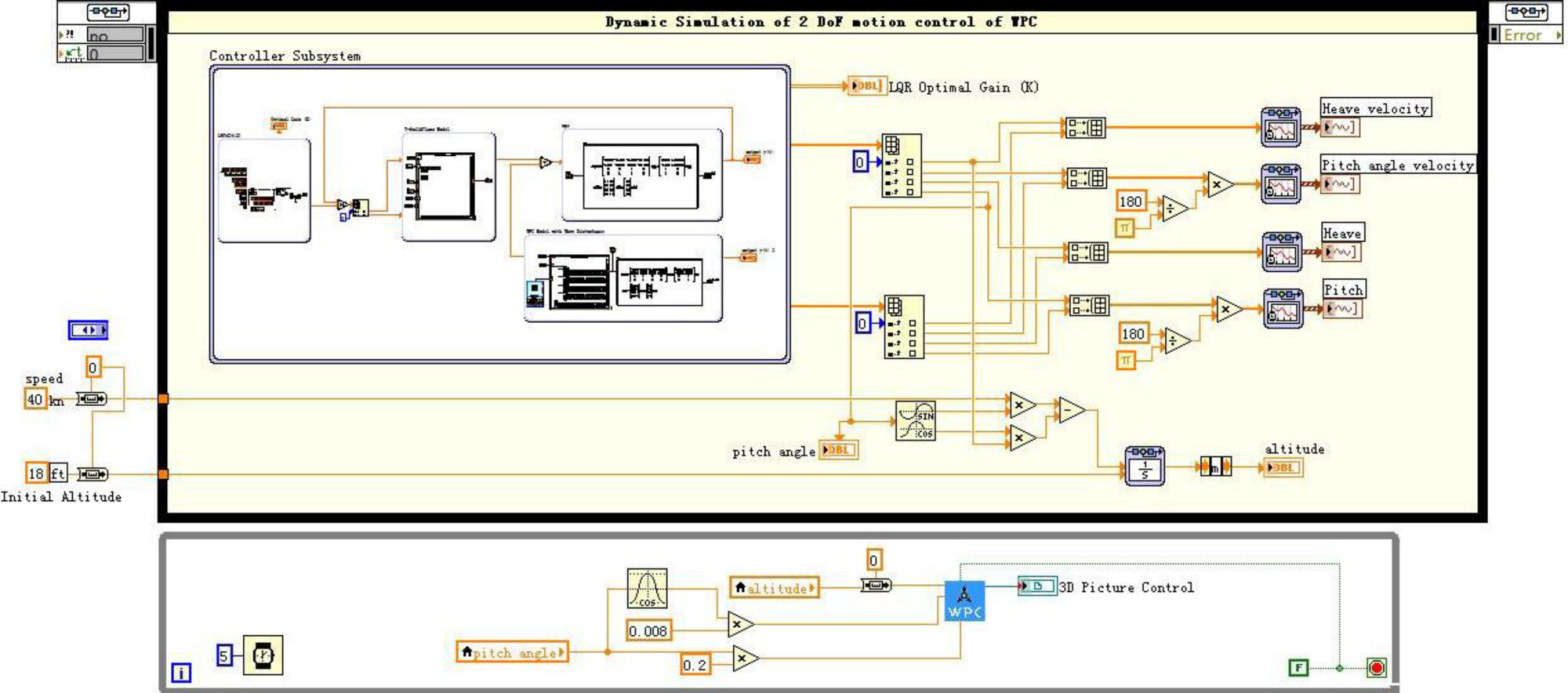
A 2 DoF WPC motion control system program.

**Fig 15:**
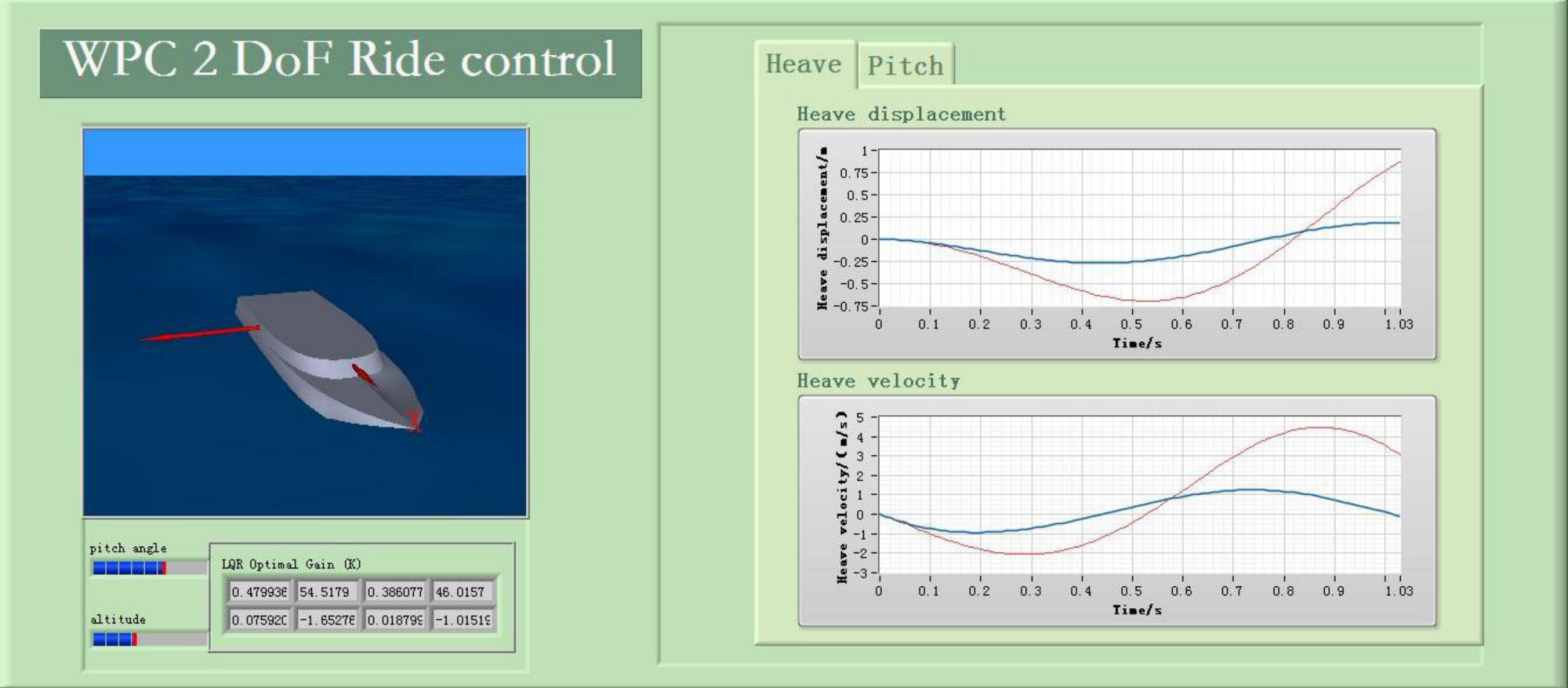
Front panel configuration of vertical control of WPC.

The LQR-GA method is designed to reduce WPC motion with the LabVIEW Real-Time (RT) module. Conveniently, the LabVIEW test bed is compatible with MATLAB simulation code to reduce the software migration workload. So some MATLAB modules have been built to carry necessary information from MATLAB to LabVIEW [41, 43]. Fig 14 shows the dynamic simulation of WPC. The outputs of the subsystem in this system include heave and pitch data with the actuators or without the actuators. The heave and pitch data connect to Data image.vi directly. Moreover, the subsystem located below this figure is a 3D animation display program. The final results are described in front panels (see Fig 15). This box involves two things: (i) It can be seen the vertical motion with an animated picture of WPC intuitively, (ii) The heave and pitch responses can be seen in chart tool during simulating. There is a control button to adjust the desired gain *K*. For instance, when the ship sailing at 40 knots and SSN4, the *K* is different from other speeds and state numbers (see Table 5).

### 4.3 Software implementation of NI CompactRIO embedded controller

The LQR-GA approach is effective when the ride control system of WPC using MATLAN and LabVIEW environment. Therefore, it is necessary to use a real embedded controller instrument instead of digital analog signal. The CompactRIO of National Instruments (NI) is used as programmable automation controller (PAC) to achieve ultra-high performance and customizable function by applying advanced embedded control and data acquisition technology. The CompactRIO embedded system with easy-to-use graphical programming tools is powered by LabVIEW exploitation environment which contains LabVIEW RT module and LabVIEW field-programmable gate arrays (FPGAs) module. A hardware description language (HDL) is designed with the logic algorithm or source code for user and is executed by LabVIEW FPGAs module to process and generate a series of synchronous analog or digital signal rapidly. Then, the data are transferred from FPGAs module to LabVIEW RT module to calculate the RT data. Finally, NI CompactRIO test bed is developed by LabVIEW exploitation environment to realize the WPC stabilization based LQR-GA [15, 42–44]. In a word, the LQR-GA method can easily implement based on MATLAB and LabVIEW FPGA tools by using the NI CompactRIO embedded controller.

**Fig 16:**
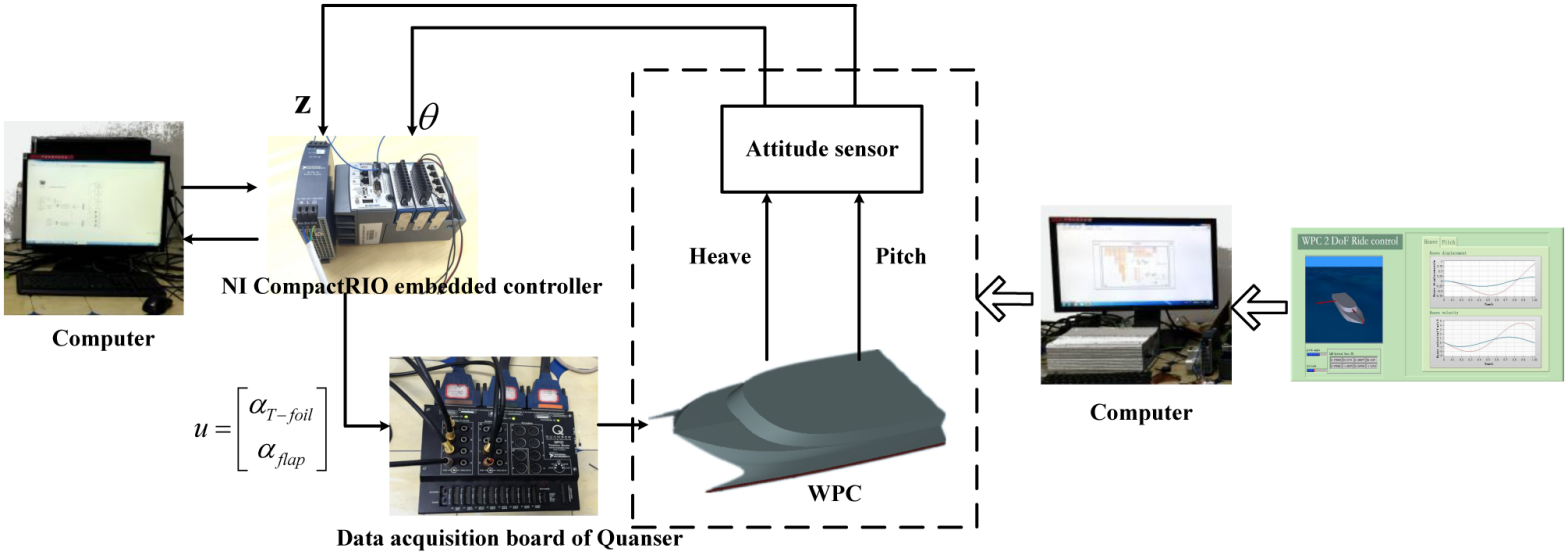
Connection diagram of overall system.

The components of the experimental devices for 2 DoF reduction of WPC are shown in Fig 16, where the heave and pitch motion of WPC are produced by computer numerical simulation using MATLAB and LabVIEW software. The NI CompactRIO embedded controller with FPGAs tool receives control signals from the computer with LQR-GA program. The control variables from above embedded controller are sent to the computer with WPC motion model through the multi channel data transmission device of Quanser. This configuration is applied to check the seakeeping of WPC with two actuators based on LQR-GA. The significant wave height changes from 1.875m to 5m in heading sea.

**Fig 17:**
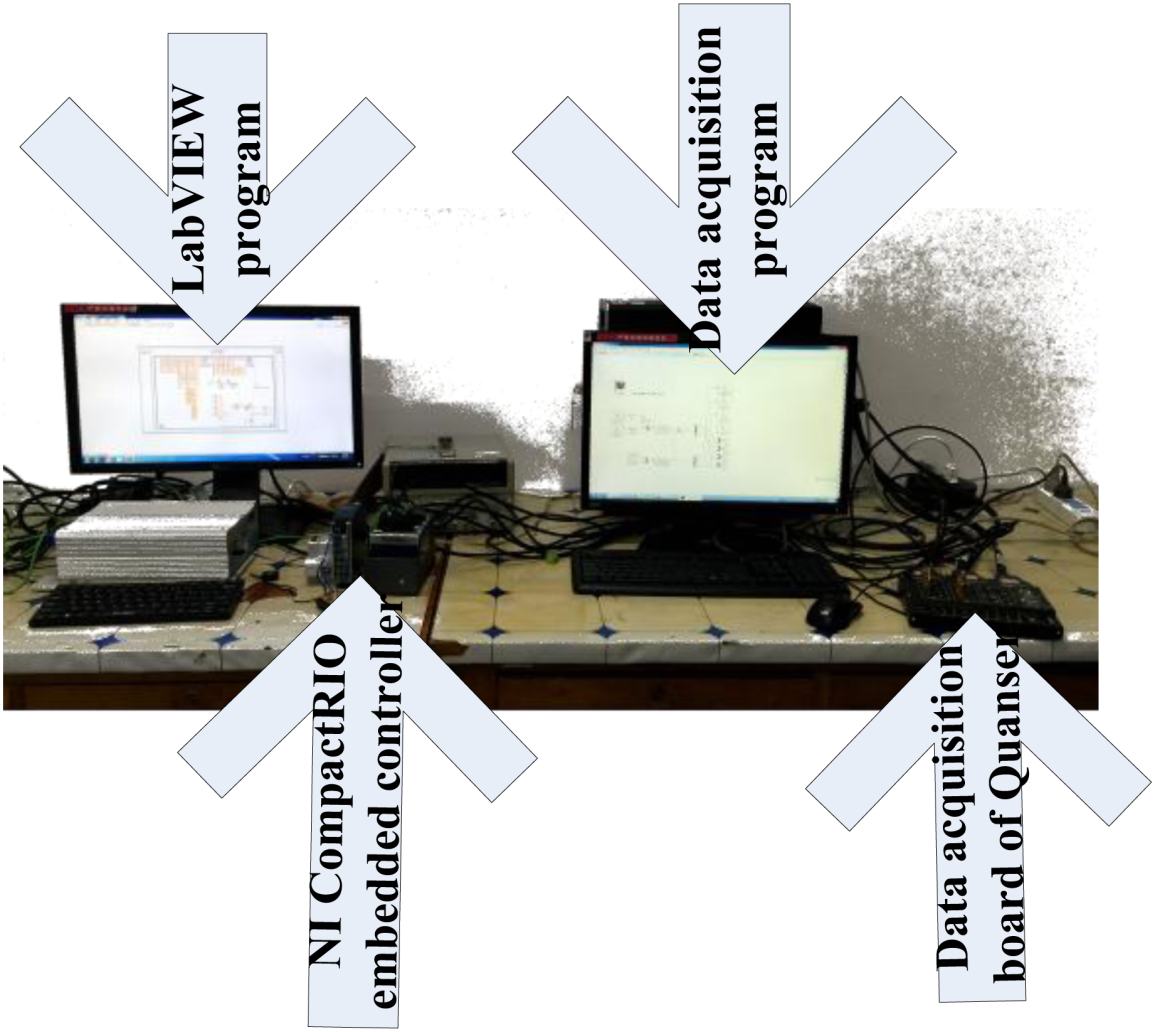
Embedded controller test plat.

**Fig 18:**
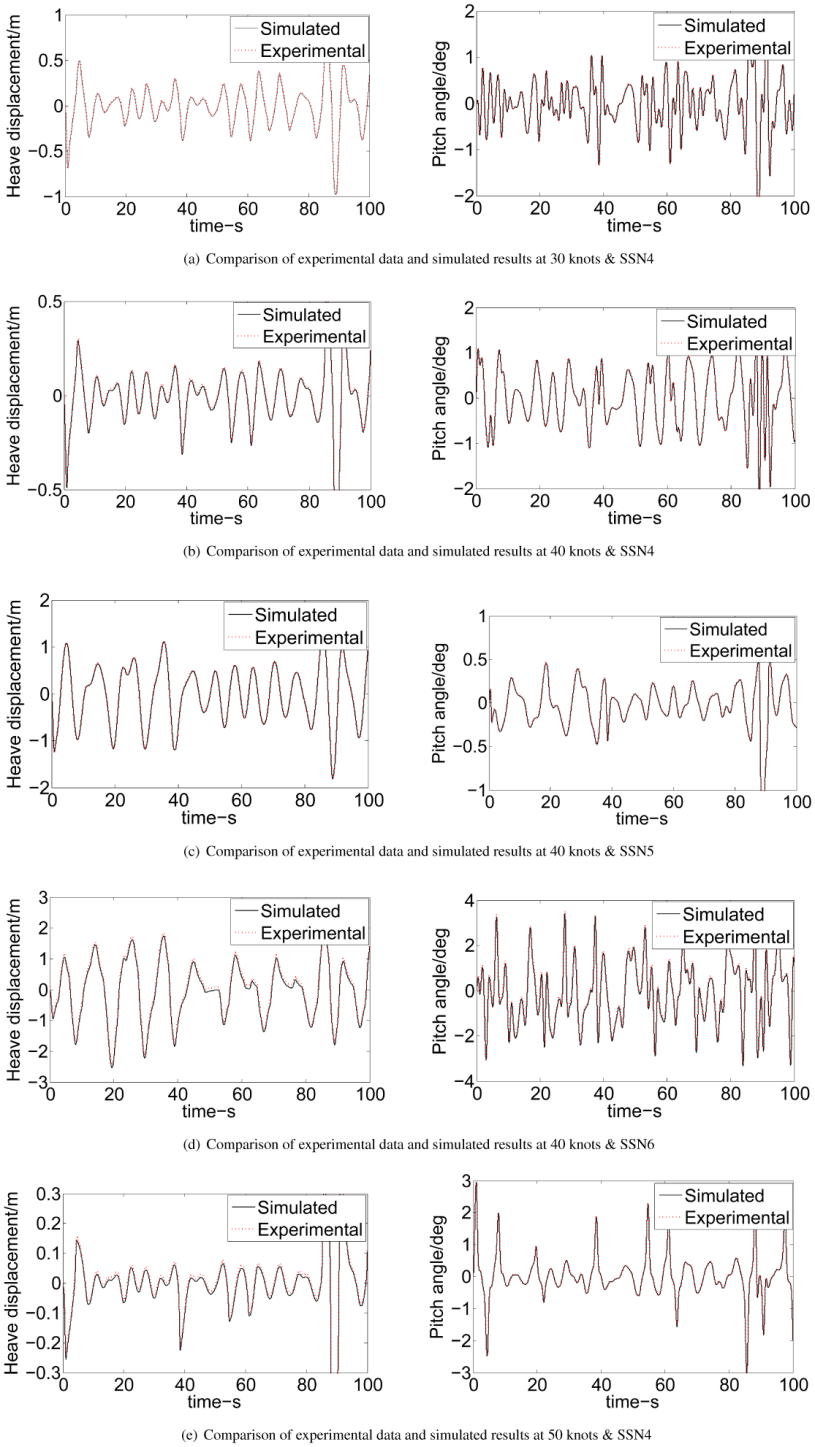
Model validation of the LQR-GA controller. (a) Comparison of experimental data and simulated results at 30 knots & SSN4. (b) Comparison of experimental data and simulated results at 40 knots & SSN4. (c) Comparison of experimental data and simulated results at 40 knots & SSN5. (d) Comparison of experimental data and simulated results at 40 knots & SSN6. (e) Comparison of experimental data and simulated results at 50 knots & SSN4.

A real controller with LQR-GA is tested instead of digital simulation of computer. While some virtual aspects (i.e., a simulation based the wave module, T-foil and flaps module and ship motion module) are built in this system and they have not been replaced yet. In Fig 17, a validated model of WPC motion is built through a computer simulation. The LQR-GA method program is downloaded in NI CompactRIO to control the numerical analog actuators on another computer. Experimental variables and data between the proposed controller and the RT processor within the NI CompactRIO embedded system could be transferred smoothly and quickly owed to built-in functions of LabVIEW. Fig 18 shows the experimental and simulated validation of the proposed controller. It can be seen that the experimental data and simulated results agree fairly well at different speeds and sea states.

## 5 Conclusion

1. A 2 DoF motion model of WPC is built, including inertia terms, damping terms, restoring terms, control terms and disturbance terms. The difficult part is to calculate the hydrodynamic parameters because of their complexity and uncertainty. Fortunately, the strip theory can solve the problem.
2. A multivariable LQR approach based GA is applied to improve 2 DoF motion of WPC. The optimal control inputs are obtained by choosing proper weighting matrices Q and R to obtain the desired gain K finally. Depending on robust search mechanisms and global optimum, GA is a better method to choose. And the stability of the proposed controller based on GA has been proven.
3. System development and simulation using MATLAB and LabVIEW environment is completed. All combinations of WPC’s speeds (30, 40, and 50 knots) and SSN (SSN4, SSN5, and SSN6) are studied to verify the correctness of proposed algorithm. Moreover, The NI CompactRIO embedded controller with easy-to-use graphical programming tools is powered by LabVIEW exploitation environment which contains LabVIEW RT module and LabVIEW FPGAs module. Consequently, the WPC motion control can be operated in real time and a 3D animation is displayed by front panel.
4. Further work with a real ship test is our next plan. We expect to our solutions lay a foundation for many researches in this field and come up with more different solutions for conducting maritime tasks.
